# Supplementary material for: DIP-MS: ultra-deep interaction proteomics for the deconvolution of protein complexes
Source: Nat Methods. 2024 Mar 26;21(4):635–47. doi: 10.1038/s41592-024-02211-y (PMC11009110; doi:10.1038/s41592-024-02211-y)
Supplement: Supplementary file 1 — Supplementary Results, Figs. 1–9, Captions for Tables 1–6, Material and Methods, Image source data of uncropped/unprocessed images and References. [file 41592_2024_2211_MOESM1_ESM.pdf]

nature methods

Article

https://doi.org/10.1038/s41592-024-02211-y

DIP-MS: ultra-deep interaction proteomics for the deconvolution of protein complexes

In the format provided by the authors and unedited

Supplementary Information

Contains Supplementary Results, Supplementary Figures 1–9, captions for Supplementary Tables 1–6, Supplementary methods, image source data for Supplementary figures and references.

Supplementary Results

Benchmark of co-fractionation-MS tools on DIP-MS data

We re-analyzed all the 3 replicates of the PFDN2 DIP-MS experiment using EPIC, PrInCE and PCprophet, using default parameters (EPIC/PrInCE) and a comparable false-discovery rate (FDR) filter to *PPIprophet* when possible (PCprophet). For EPIC/PrInCE no comparable FDR assessment is possible. It is important to point out that these comparisons are inherently problematic because the respective tools were developed with different objectives in mind. In these comparisons, we focused on PPIs because both, PrInCE and EPIC utilize third-party software for graph partitioning and they further prune the graph using functional data, which might obfuscate raw prediction performance. As PCprophet does predict complexes directly and not protein interactions per se, we decided to derive networks from the predicted complexes assuming fully connected complexes. It is important to keep the latter point in mind as it results in a large number of interactions for a single complex as the software needs to predict only 1 positive (the complex) versus all the single interactions, which inflates true positives for specific analysis.

We started by evaluating the total number of protein-protein interactions that were identified as a proxy for the utilization of co-elution data. In total, EPIC and PrInCE predicted ~20'000 unique PPIs across the PFDN2 DIP-MS dataset while *PPIprophet* resulted in 3873 PPIs in total (using only high-confidence interactions plus CRAPome filtering of 10% for both nodes (absolute of (ProtA CRAPome frequency – ProtB CRAPome frequency)). PCprophet predicted ~10'000 interactions, but it is important to note that large complexes like the

ribosome or the proteosome, will results in many PPIs, hence at the PPI-level this number is likely inflated by the assumption of fully connected complexes mentioned above (Supplementary Fig. 1A).

We then categorized the total number of PPIs detected by the respective tools, depending on whether the interactions were present in STRING or whether there was no prior interaction evidence for them and then calculated the ratio of each of these two classes to the total per each tool. In this analysis, a higher percentage of overlapping STRING interactions suggests a lower false-positive rate, irrespective of the total number of interactions identified. The large number of interactions covered by EPIC or PrInCE did however not translate in a greater recovery of reported interactions in STRING with only 29% (5629/19247) of the total PPIs from PrInCE being previously reported, potentially suggesting a greater FDR for this tool. EPIC identified 47% of previously reported PPIs (9496/20143), on par with *PPIprophet* (47%, 1876/3836) as demonstrated in Supplementary Fig. 1B. PCprophet in this analysis outperformed the other tools (92% of the derived interactions are present in STRING, 2829/3061) due to the over-representation of large complexes (i.e. ribosome, proteasome, etc.) which results in a large number of true interactions when converting complexes predicted by PCprophet into protein-protein interactions.

We next focused on evaluating the recovery of known interactions for the canonical PFD and the PAQosome complexes. To achieve so, we extracted for every tool all the interactions for every PFD components (PFDN1, PFDN2, PFDN4, PFDN5, PFDN6 and VBP1) and PAQosome components (PFDN2, PFDN6, URI, UXT, POLR2E, WDR92, RUVBL1, RUVBL2, ASDURF, PDRG1 and PIH1D1) and separated them into true positive (TP) and false positive (FP) interactions using our manually curated list of reported PPIs and complexes of PFD, PFDL and PFDL-containing PAQosome (Supplementary Table 4).

These two classes were then used to assess the positive predictive value or precision (defined as TP / (TP + FP)) for the various tools at the complex-level as the goal of DIP-MS is to reliably identify complexes from their constituent interactions. When performing this analysis, we identified *PPIprophet* as the tool having the greatest positive predictive value for the PFD

interactions (94% precision) and the PAQosome (60% precision) as shown in Supplementary Fig. 1C.

Overall, the results of these benchmarking analyses suggest a superior performance of *PPIprophet* over other tools to analyze DIP-MS datasets in terms of specificity of recovery of specific interactions of the PFD and PAQosome complexes from affinity-purified fractionated samples. It needs to be noted that different tools have been developed for different purposes. The DIP-MS data is substantially different from a global co-fractionation MS dataset as it encompasses few proteins with large number of peaks and abundances spanning several orders of magnitude. Hence, tools relying on global metrics such as global correlation, Euclidean distances etc., exemplified by EPIC/PrInCE, will be at a disadvantage by utilizing a single feature to classify highly convoluted protein-coelution profiles. This is exemplified by the lower precision of PFDN/PAQosome specific interactions which are proteins known to be in multiple peaks and at different abundances. On the other hand, the computational complexity from global co-fractionation MS datasets cannot be readily handled by *PPIprophet* due to the need to construct a global matrix (N x N) which poorly scales with increased protein numbers.

Recovery of PFD, PFDL and PAQosome in DIP-MS vs. coelution database

In the SEC-MS dataset the PFD complex was identified from the profiles of coeluting proteins, whereas the PFDL complex could not be identified compared to both DIP-MS experiments (Supplementary Fig. 2A). Comparison to 32 coelution studies of human cell extracts showed that on average 3 out of 6 PFDL subunits were recovered with only 2 studies recovering all 6 subunits (Supplementary Fig. 2B). This further supports that the combination of enrichment and fractionation boosts the depth and resolution of co-fractionation data to recovery low abundant proteins.

Orthogonal evidence of DIP-MS derived PPIs by *in vivo* proximity interaction datasets

To investigate how proximity ligation experiments compare to the presented DIP-MS approach, we assembled a dataset of BioID interactions (see Supplementary Methods). We started by evaluating the overlap of the 424 proximity PPIs with the 2’395 PPIs obtained from DIP-MS experiments of PFDN2 and UXT. In total 78 PPIs (18% of the BioID dataset) overlapped with our DIP-MS derived protein-interaction network (Supplementary Fig. 3A). Of the overlapping PPIs, 66 were already present in the manually curated list of reported PPIs obtained with other techniques, whereas the other 12 PPIs were exclusively reported by biotin proximity ligation experiments. A large number of PPIs were only found within each dataset, which indicates that these two approaches are orthogonal to each other.

We investigated further the 78 PPIs for their relevance and biological functions. Despite the small overlap of PPIs, 20 protein interactions between PAQosome core-subunits were validated by proximity ligation data. In addition, protein interactions with all 16 core subunits have been identified by both techniques, resulting in a fully connected protein interaction network (Supplementary Fig. 3B). Within the PPI network, well-established PAQosome and canonical Prefoldin interactors are covered with multiple interactions. For the canonical prefoldin, a tubulin subunit was identified to interact with multiple PFD subunits in both datasets. Further, multiple PPIs between the PAQosome und known interactors such as RNA polymerase subunits (10 subunits, 16 PPIs) and PP1-gamma subunits (2 subunits, 16 PPIs) were recovered by DIP-MS and BioID. Of note, also the link between the PAQosome and the dynein-dynactin complex was mapped by both methods with 7 PPIs to 3 dynein-dynactin subunits.

These results show that PPIs recovered by DIP-MS are found by orthogonal technologies such as in vivo proximity ligation. The BioID data only overlap partially with DIP-MS data, which is indicative that the different interaction methods are orthogonal to each other as reported when BioID was compared to AP-MS (Lambert JP et al., 2015, Proteomics).

Structural modelling of PFDL complex

Like for the PFD and PFD homologous complex, we aimed to model the structure of the PFDL complex. Structural prediction of the PFDL complex performed poorly (not shown) due to C-terminal tail of URI1, for which the AlphaFold2 pLDDT residue score was low (Extended data Fig. 6E). As scores below 50 pLDDT are associated with intrinsically disordered regions (IDRs), we employed flDPnn [1] to predict IDRs within the C-terminal tail of URI1 (Extended data Fig. 6E) and identified two IDRs in the URI1 (aa1-23 and 223-431). The flDPnn model predicted two distinctive protein binding regions which is in agreement with previous results [2], regarding URI1 binding POLR2E. We shortened the URI1 sequence by removing the IDRs region and modeled the PFDL-complex (PFDN2, PFDN6, PDRG1, ASDURF, UXT and URI aa 24 - 222) for which we obtained a complex, with a weighted confidence score of 0.75 (Extended data Fig. 6F).

PAQosome coeluting proteins and quantitative comparison between DIP-MS experiments

From the assigned 107 proteins which show coelution with the PAQosome, PFDL and the adaptor complex, the majority was grouped to different complexes and could be linked through literature evidence (Fig. 6).

For 21 grouped client-proteins, no direct PAQosome-interaction was previously reported in literature and for 4 of them (AAR2, GPN3, RSL1D1, TOP1) we recovered PPIs within our reciprocal AP-MS dataset with PAQosome components such as RPAP3, PIH1D1 and UXT. In a recent study the PAQosome was linked to ribosomal maturation via transient interaction between the unphosphorylated subunit RPAP3 with ribosomal proteins (Extended Data Figure 1) [3]. Despite low abundance of this interaction, we confirmed multiple hits from this study including ribosomal protein RSL1D1, thereby providing evidence of a potential role of

the PAQosome in ribosomal maturation. It is important to point out that most of these peaks represent fractional amounts of the total protein signal as many proteins moonlight into different assemblies or show a CCI between a client-complex and the PAQosome.

Next, we compared the abundance of the recovered PAQosome clients in the UXT and PFDN2 DIP-MS. The PAQosome core-subunits were fully identified in both DIP-MS experiments (compare Extended data Fig. 2). 102 clients (or 95%) were quantified in the PFDN2 DIP-MS versus 72 clients (or 68%) in UXT DIP-MS experiment (Extended data Fig. 9A, B). We found that 31 clients were enriched with at least 0.5 log2FC in the UXT DIP-MS and 54 clients in the PFDN2 DIP-MS experiment (log2FC UXT/PFDN2 DIP-MS) (Extended data Fig. 9C). These indicates that the two DIP-MS experiments overlap across large-parts of the recovered clients, allowing to complement each other. The specificity of the in the DIP-MS experiment coeluting proteins can further be demonstrated via the specific enrichment of PAQosome clients in the AP-MS using PAQosome subunits as baits compared to the enrichment observed with Prefoldin specific bait proteins (Extended data Fig. 9D).

Supplementary Figures

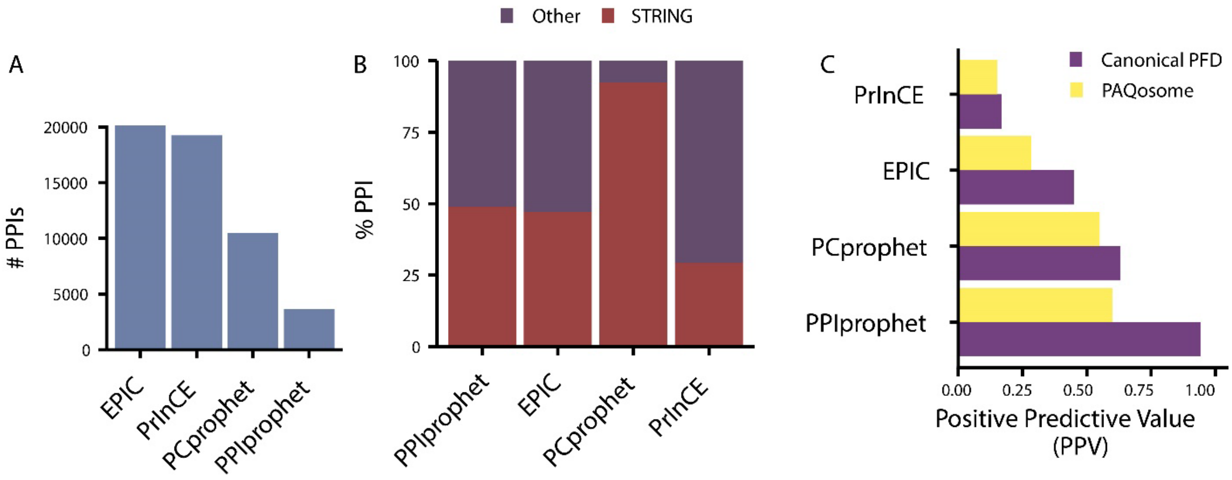

Supplementary Fig. 1. Benchmark of co-fractionation MS tools on DIP-MS data. A. Barplot illustrating the total number of PPIs identified by the various tools. **B.** Recovery of known interactions from STRING (red bar) over the total number of interactions identified, expressed as percentile for the different tools employed. **C**. Positive predictive value (i.e. precision, X-axis) for the canonical PFD (purple) and the PAQosome complex (yellow bar).

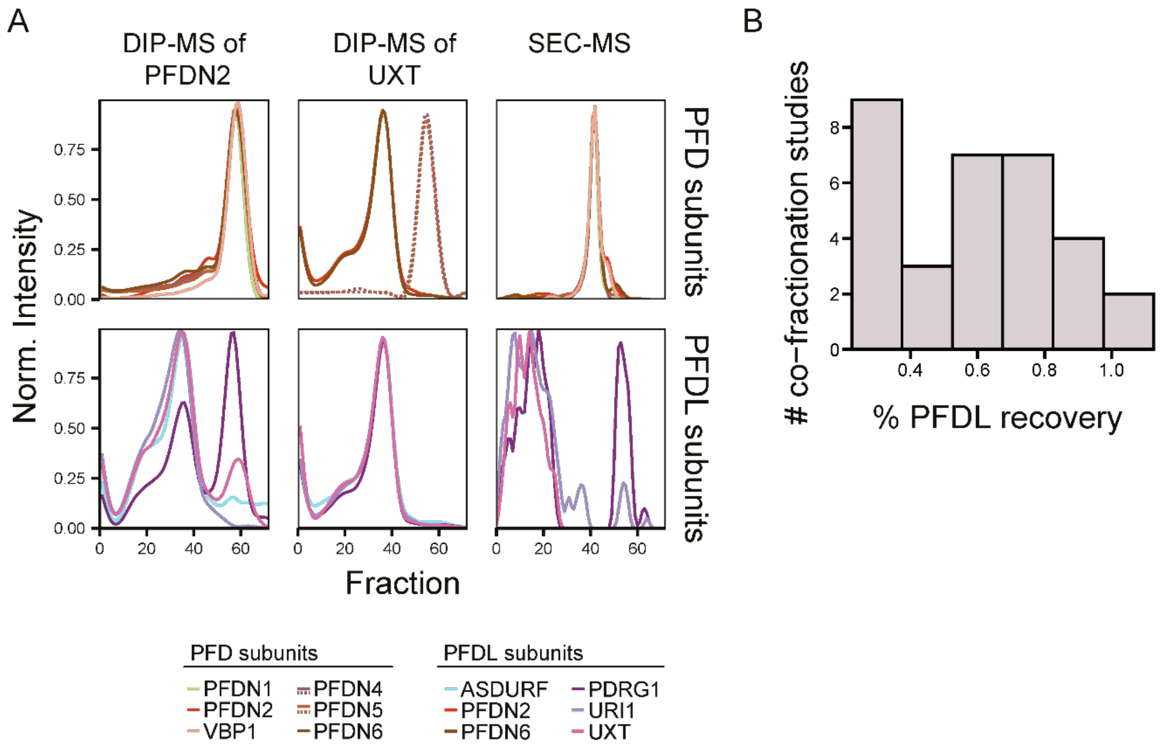

Supplementary Fig. 2. Comparison of DIP-MS profiles to publicly available SEC-MS datasets for the PFD and PFDL complex. A. Coelution profile for PFD and PFDL subunits separated by complex **[4]**. UXT DIP-MS quantified a coelution profile of PFDN4 and PFDN5 (indicated with a dashed line) at the canonical PFD peak. They do not show any coelution with other PFDL subunits and the MS2 protein abundance) compared to the PFDN2 signal at the PFDL complex coelution group peak is 3 orders of magnitude lower (FC for PFDN4 and PFDN5 at PFD peak vs PFDN2 at PFD peak 1.36×10^{-3} respectively 3.46×10^{-3}). We marked them as likely contaminants in the DIP-MS UXT with a dashed line. **B.** Recovery of PFDL subunits in coelution profiling studies of human cells reported in Skinnider et al. [5].

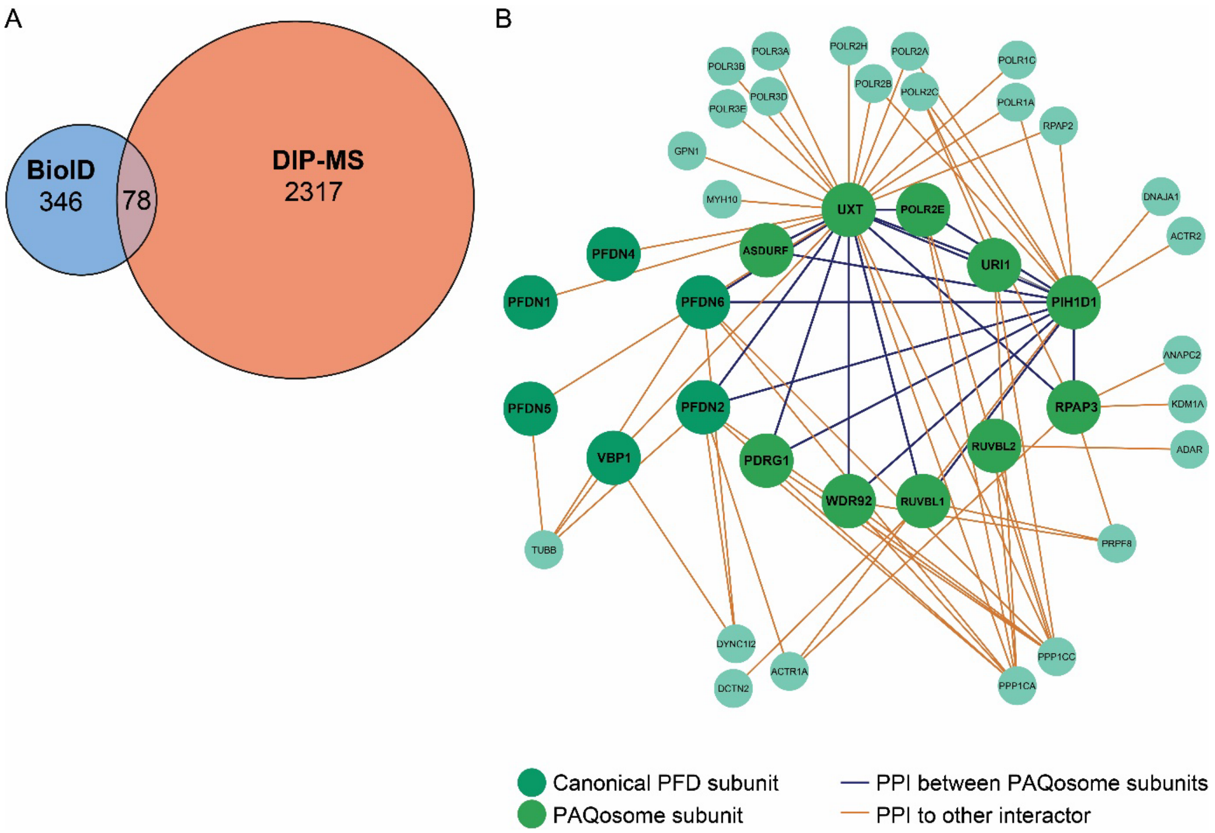

Supplementary Fig. 3. Comparison of DIP-MS obtained PPIs to in-vivo proximity ligation (BioID) derived PPIs. A. Venn Diagram of DIP-MS derived PPIs overlapped with proximity interactions obtained by an assembled dataset of proximity interactions. **B. Interaction** network derived from the 78 overlapping PPIs. PFD complex subunits are colored in dark green and PFDL and PFDL-containing PAQosome are green. The network contains 41 nodes with 78 edges. Edges between PAQosome subunits are colored in blue, edges to other interactions in orange.

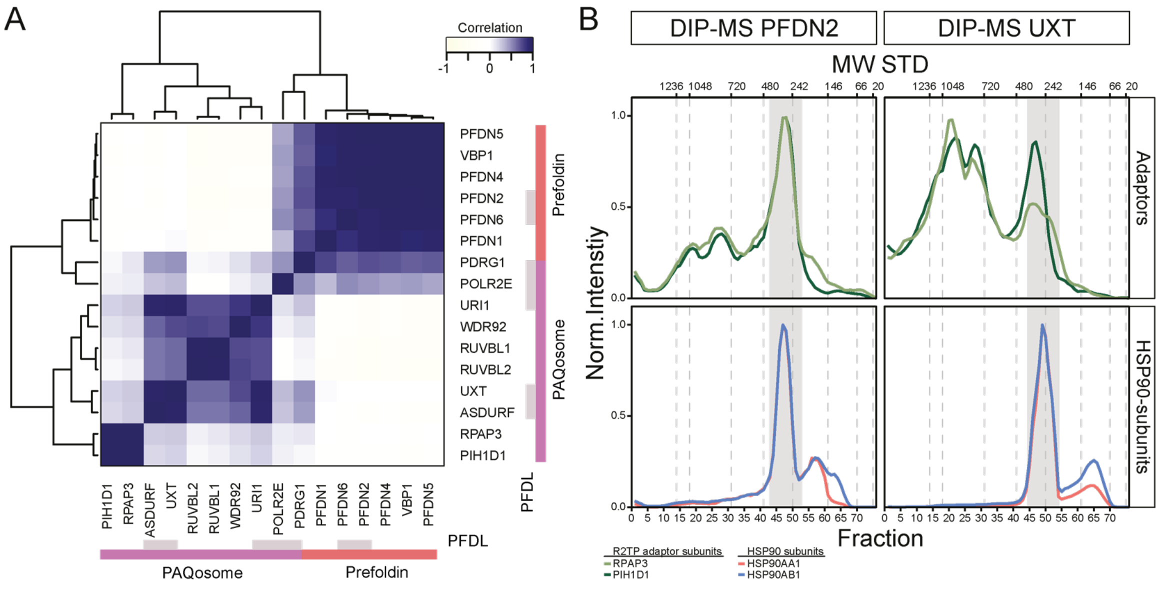

Supplementary Fig. 4. Clustering of PFD and PAQosome subunits and coelution of RPAP3:PIH1D1 with HSP90 subunits. A. Heatmap representing the hierarchical clustering using Ward's distance for PFD (Red) and PAQosome (grey) subunits from the PFDN2 DIP-MS experiments. Cell color represents the Pearson's correlation. **B.** Coelution group of RPAP3:PIH1D1 (R2TP adaptors) with HSP90 subunits identified in the PFDN2 and UXT DIP-MS experiment at an apparent MW 356 kDa or Fraction 45. The intensity shows the smoothed average MS2 protein intensity normalized to the maximum protein intensity across all gel-slices.

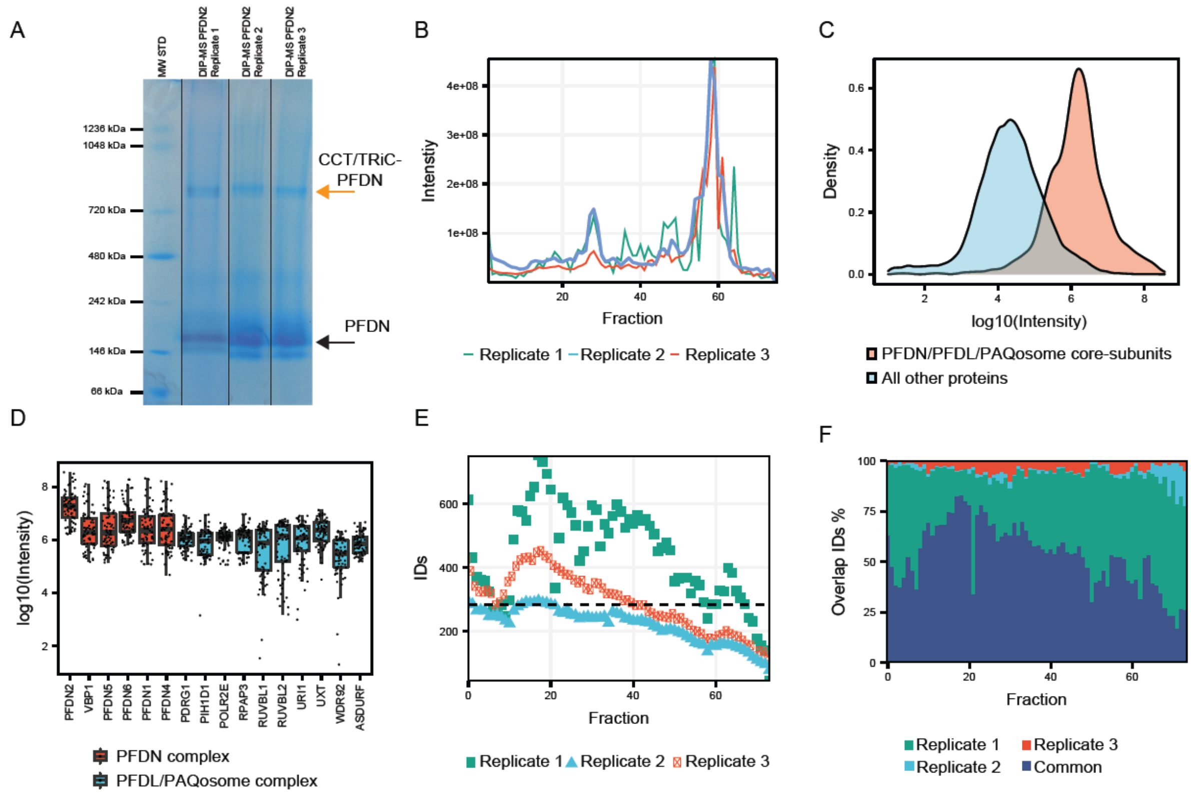

Supplementary Fig. 5. Reproducibility of DIP-MS for PFDN2 across replicates. A. Blue native-PAGE of PFDN2 replicates after staining with SimplyBlueTM SafeStain. First lane contains the molecular weight (MW) standard. The PFD complex is visible as a band at an apparent MW of ~150 kDa or Fraction 59 (band indicated by black arrow). The visible band at an apparent MW of ~800 kDa or Fraction 28 corresponds to the chaperone complex CCT/TRiC together with PFDN complex (band indicated by orange arrow) (n=2 biologically independent blue native-PAGE separations). The uncropped and unprocessed images of the blue native-PAGE images are provided as image source data 1. **B.** MS2 based LFQ TIC of the PFDN2 replicates across the 74 fractions after peak realignment (n=3 independent biological experiments). **C.** Density plot showing the abundance of 16 PFD and PFDL/PAQosome core-subunits over all fractions (red distribution) against all other quantified proteins (blue distribution). **D.** Distribution of protein abundances (logged MS2 intensities) across all fractions for 16 PFD and PFDL/PAQosome core-subunits, solid line represents the median, box limits show the IQR and its whiskers 1.5 x IQR. Each point represents the summed total intensity of one core-subunit per each fraction/slice (n=74 maximum number of fractions/slices for each subunit, obtained from n=3 biological independent replicates). **E.** Scatterplot showing the number of identified proteins per fraction. Dashed line shows the mean. **F.** Barplot representing the number of unique and shared proteins identified per fraction across the three PFDN2 DIP-MS experiments.

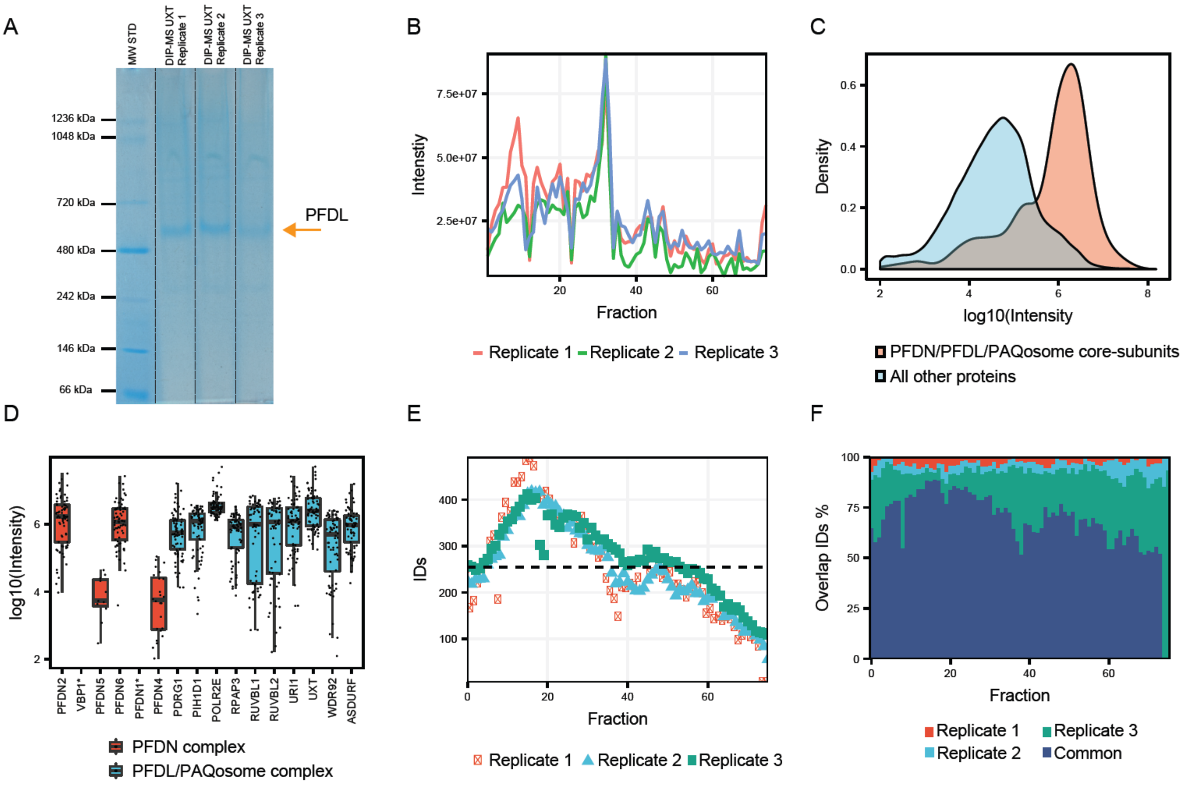

Supplementary Fig. 6. Reproducibility of DIP-MS of UXT across replicates. A. Blue native-PAGE of UXT replicates after staining. The molecular weight standard (MW STD) was loaded in the first lane. The PFDL module migrates at an apparent ~MW weight of ~500 kDa and peaks in fraction 38 (band indicated by orange arrow) (n=1 blue native-PAGE separation). The uncropped and unprocessed images of the blue native-PAGE image are provided as image source data 2. **B.** TIC of the three replicates across the 76 fractions after peak realignment (n=3 independent biological experiments). **C.** Density plot showing the abundance of 16 PFD and PAQosome core-subunits over all fractions (red distribution) against all other quantified proteins (blue distribution). **D.** Distribution of protein abundances (MS2 intensities) across all fractions for 16 PFD and PAQosome core-subunits, solid line represents the mean, box limits shows the IQR and its whiskers 1.5 x IQR. Each point represents the summed total intensity of one core-subunit per each fraction/slice (n=76 maximum number of fractions/slices for each subunit, obtained from n=3 biological independent replicates. Not recovered PFD-subunits VBP1 and PFDN1 are marked with an asterisk '*'). **E.** Scatterplot showing the number of identified proteins per fraction. Dashed line shows the mean. **F.** Barplot representing the number of unique and shared proteins identified per fraction across the three UXT DIP-MS experiments.

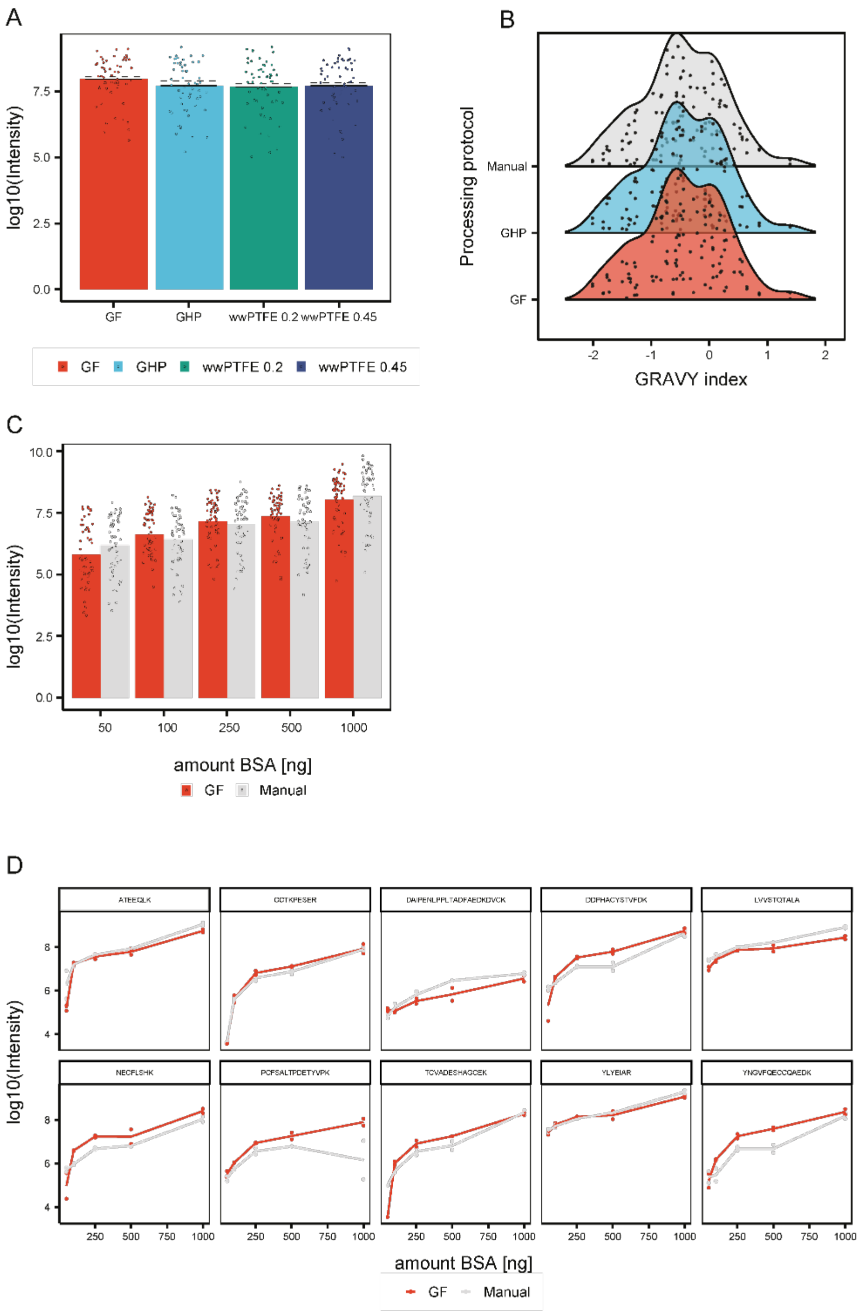

Supplementary Fig. 7. Optimization high-throughput preparation of Gel-slices. A. Barplot showing the recovery of 500 ng of BSA using different filter materials. Y axis shows the precursor ion intensity. Each point represents the average signal intensity of one peptide (n = 27 peptides per condition). The dotted line corresponds to the median, the solid line represents the mean (n=3 biologically independent samples per filter plate). **B.** Density plot of GRAVY index per peptide across Glass Filter (GF) and GHP membrane 96-wellfilter plates versus manual in-gel preparation of gel-slices (1000 ng loading, n = 2 biologically independent samples). **C.** Bar plot showing the recovery of BSA peptides between manual processing (grey bars) or filter-based processing. X axis represents the amount of BSA processed. Y axis shows the correspondent peptide-level intensity in a log10 scale. Each data point is the mean of a single peptide for the duplicate injections (n = 2 biologically independent samples). **D.** Recovery of BSA peptides with glass filter (GF) plate (red) against manual in-gel preparation (grey) of 10 BSA peptides using different amounts (50, 100, 250, 500, and 1000 ng). The curve shows mean of the duplicates per each condition (n=2 biologically independent samples).

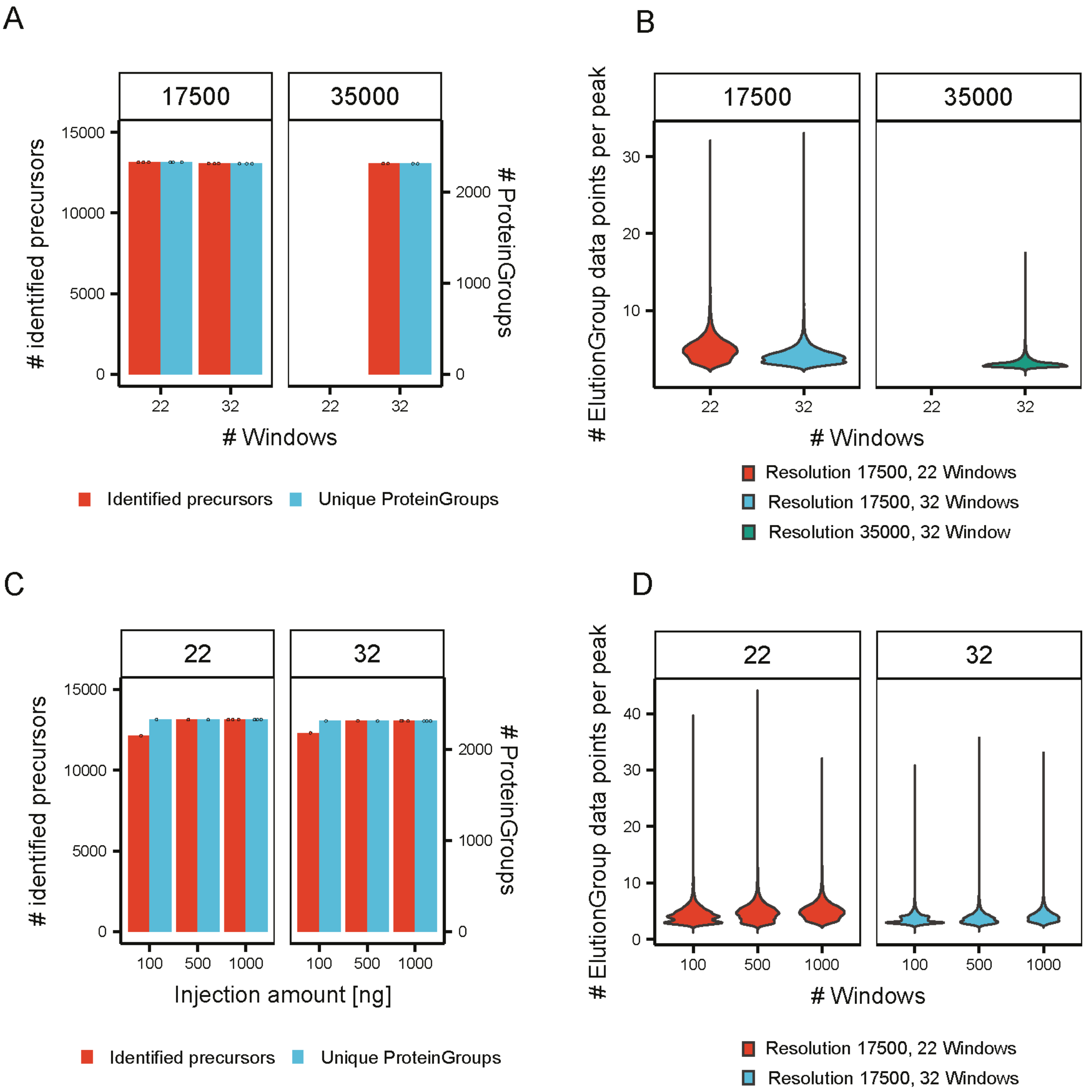

Supplementary Fig. 8. DIA-method optimization for 24 minutes gradients for DIP-MS. A. Barplot showing the mean of uniquely identified peptides (left Y axis, red bars), and uniquely identified protein IDs (right Y axis, blue bars) for 22 vs. 32 variable windows at 17’500 and 35’000 resolutions of 1000 ng of HEK293 WT (ATCC collection) lysate. The barplots show the average (n=3 biologically independent experiments at 17’500 resolution and n=2 biologically independent experiments at 35’000 resolution). **B.** Violin plots of fragments data points per peak. Different colors show different resolution and number of DIA windows (all with n=3 biologically independent experiments). **C.** Dilution series of HEK293 WT (ATCC collection) from 100 to 1000 ng showing the comparison between the 22 vs 32 windows DIA schemes. Unique peptides and protein groups are showed as Y axis on the left and right respectively (n=3 for 1000 ng, n=1 for 100 and 500, biological independent experiments). **D.** Violin plot representing the points per peaks from DIA methods with 22 or 32 variable windows at 17500 MS2-resolution for different amounts of HEK293 WT lysate (n=3 for 1000 ng, n=1 for 100 and 500, biological independent experiments).

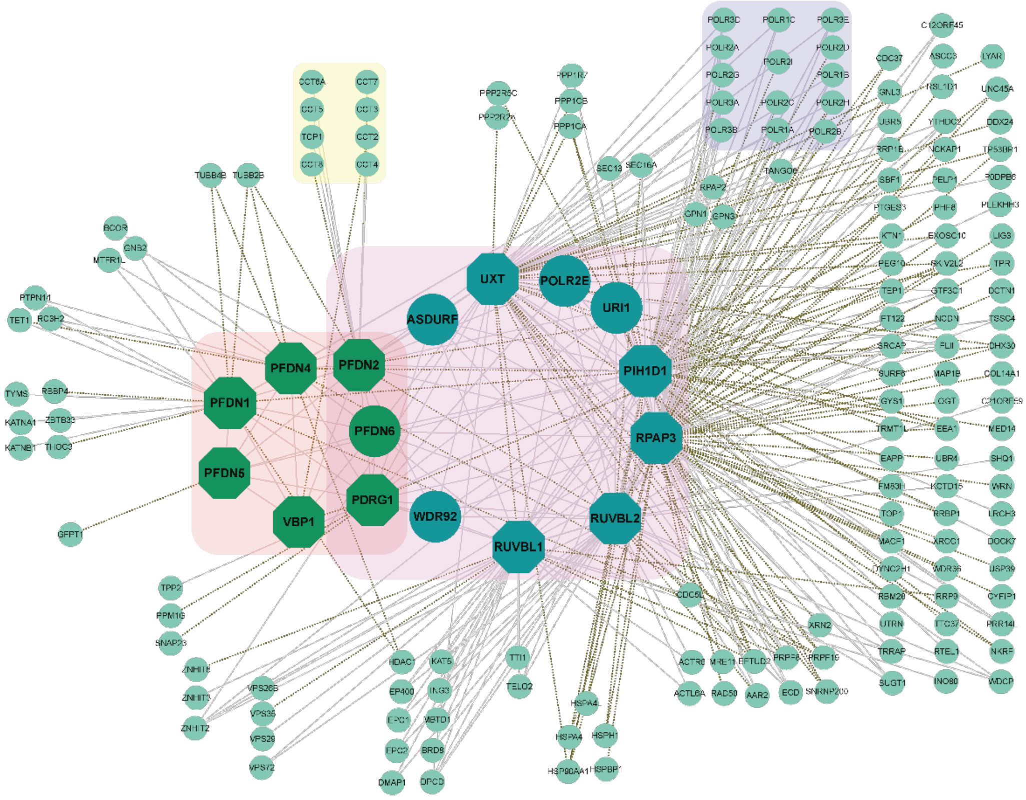

Supplementary Fig. 9. Interaction network of Prefoldin and PFDL containing PAQosome (R2TP/PFDL) subunits identified by reciprocal AP-MS. Interactome obtained by AP-MS using the PFD, PFDL and PAQosome subunits as baits. All baits are depicted as octagonal node shape. PFD subunits are in light green (red background) and PAQosome subunits in cyan (purple background). RNA polymerase subunits (blue background) and CCT/TRiC complex subunit (yellow background) are grouped together. Grey edges represent high-confidence interactions (Log2FC ≥ 5 and Saint score ≥ 0.99) while dashed edges are medium confidence interactions (Log2FC ≥ 2 and Saint score ≥ 0.95 and <0.99).

Supplementary Tables

- Supplementary **Table 1**: Core-subunits of PFD, PFDL and PAQosome.
- Supplementary **Table 2**: Prediction performance of PPIprophet.
- Supplementary **Table 3**: List of AP-MS baits used for validations within this study.
- Supplementary **Table 4**: List of reference datasets. Curated list of interactors of PFD, PFDL and CCT/TRiC subunits derived from literature.
- Supplementary **Table 5**: Literature derived complex associations of PFD, PFDL and PAQosome.
- Supplementary **Table 6**: Resources and materials.

Supplementary Material and Methods

Expression constructs

For the affinity purification experiments, N-terminal or C-terminal tagged-bait proteins were generated. Twin-Strep and hemagglutinin (SH) tagged expression constructs were generated with LR clonase (Invitrogen) using an in-house designed destination vector (pcDNA5-FRT-TOSH-GW) [6] and pDONR223 vectors of the Gateway-compatible human ORFeome collection (hORFeome v5.1, Open Biosystems) . A list of all bait proteins used in this study can be found in Supplementary Table 3. The sequences of all clones for stable cell line generation were checked by sequencing.

Generation of stable cell lines

To generate stable cell lines for inducible expression of SH-tagged bait protein [6], Flp-In HEK293 T-REx cells were co-transfected with the expression vector containing the tagged-bait construct and pOG44 vector (Invitrogen) for expressing the Flp-recombinase using jetPrime (Polyplus) following the manufactures protocol. Cells which had undergone recombination were selected for 2-3 weeks in the presence of15 μg/mL Basticidin C and 100

*µ*g/mL hygromycin 48 h after transfection , before expression of the tagged-bait was controlled by affinity purification.

Cell culture

Flp-In HEK293 T-REx cells ectopically expressing strep-HA tagged bait protein [6] were cultured in DMEM (4.5 g/L glucose, 2 mM L-glutamine) supplemented with 10% FBS, 1% Pen-Strep, maintained at 37 °C and 5% CO_2 on a 15 cm Nucleon dish (Thermo-Fisher) to reach 80% confluence before expression of the bait-protein was induced (1 *µ*g/mL doxycycline for 24 h). For harvesting, plates containing 2×10^7 cells were placed on an ice-cold rack and the media was removed. Cells were flushed off with 5 mL of PBS pH 7.4 (Gibco) and pelleted at 4 °C at 300×*g*. The supernatant was removed for subsequent shock freezing of the cells in liquid nitrogen and storage at −80 °C.

Protein purification for AP-MS experiments

For each AP a cell pellet derived from four 150 mm culture dishes was lysed in 4 mL ice-cooled HNN-lysis buffer (50 mM HEPES, 100 mM NaCl, 50 mM NaF, pH 7.4) supplemented with protease inhibitor cocktail, 1 mM PMSF, 400 nM Vanadate, 1.2 *µ*M Avidin and 0.5% NP40. Lysed cells were rested on ice for 10 minutes before clarification by centrifugation at 16’000×*g* at 4 °C for 20 minutes. Strep-Tactin Sepharose beads were equilibrated with 2 CV of HNN-lysis buffer and resuspended at 50% (v/v) slurry in HNN lysis buffer. The lysates were incubated with 100 *µ*L of beads slurry on an end-over-end tube rotator at 12 rpm for 45 minutes. Cleared lysate was loaded on Bio-Spin chromatography columns. Beads were washed twice with 1 mL ice-cooled HNN-lysis buffer and three times with 1 mL of HNN buffer without supplements. Purified complexes were eluted three times 200 µL 2 mM Biotin buffer. To precipitate enriched proteins, trichloroacetic acid (TCA) was added to 25% (v/v) and samples were incubated overnight at -20 °C. The proteins were pelleted by centrifugation at 16’000×*g* for 20 minutes at 4 °C and the supernatant was removed. The pellet was washed

twice with 900 µL -20 °C cold Acetone to remove remaining TCA. After removal of the supernatant, pellets were dried under vacuum for 10 minutes.

MS-sample processing of reciprocal AP

The protein pellet was resuspended in in 30 µL of 8 M Urea dissolved in 100 mM Ammonium bicarbonate (ABC) pH 8.8 and incubated at 1000 rpm at 25 °C. Urea concentration was lowered to 0.8 M by addition of 250 µL of 100 mM ABC. Next, TCEP was added to a final concentration of 5 mM and the samples were incubated for 30 minutes at 37 °C and 1000 rpm. Ioadacetamide (IAA) was added to 10 mM final concentration for alkylation of cysteine residues and the samples were incubated in the dark for 45 minutes at 25 °C at 1000 rpm. Proteins were digested by addition of 1 µg of Trypsin and incubated overnight at 37 °C at 600 rpm. Proteolysis was quenched by addition of 0.05% TFA to reach ≈ pH 2. Peptides were purified over a C18 microspin column. Briefly, C18 columns were activated by washing twice with 200 µL 100% MeOH and twice with 200 µL 80% ACN in 0.1% TFA. Columns were equilibrated trice with 200 µL 2% ACN, 0.1% TFA in H_2O, before loading the samples. The bound peptides were washed 5 times with 200 µL 2% ACN, 0.1% TFA in H_2O before elution with a 100 µL of 50% ACN in 0.1% TFA. Peptides were vacuum dried and stored at -80 °C.

Data acquisition of reciprocal AP samples

The AP-MS samples were acquired in DDA-mode on a Q Exactive™ Plus Hybrid Quadrupole-Orbitrap™ Mass Spectrometer (ThermoFisher Scientific) interfaced with an Easy NanoLC 1000 HPLC equipped with an autosampler (ThermoFisher Scientific). The dried peptides were dissolved in 20 µL of buffer A (2% ACN and 0.1% FA in H_2O) with 1:50 (v/v) iRT peptides (Biognoysis). For each sample, 2 µL were injected, and the peptides were separated by reverse-phase chromatography on a high-pressure liquid chromatography (HPLC) column (75 µm inner diameter, New Objective) manually packed with 15 cm of C18 beads (ReproSil-Pur 120 Å, C18-AQ 1.9 µm, Dr. Maisch GmbH) with a 10 µm fused silica tip emitter. Peptides

were separated over a 90 min gradient from 5% to 30% buffer B (95% ACN in 0.1% FA in H_2O) in Buffer A followed by step increase from 30% to 90% of buffer B in 7 minutes and a column clean-up by holding the gradient for 8 minutes at 90% buffer B. The capillary voltage was set to 2.2 KeV and 250 °C. The MS was operated in positive mode, with MS1 and MS2 scans collected in the Orbitrap analyzer (OT/OT). MS1 resolution was fixed at 70'000 at 200 *m/z* while the resolution for fragment ions was set at 35'000. The MS1 scan range was set from 350 to 1650 *m/z* with a parent ion isolation window of 1.5 *m/z*. The default charge state was set to +2, and unassigned charge states and +1 were excluded from fragmentation. The AGC target for MS1 scans were set to $1e^6$ with a maximum accumulation time of 54 ms. The 15 most abundant precursors per scan were fragmented with high-collisional induced dissociation (HCD) using a normalized energy of 27% and were subsequently excluded for 15 seconds. Fragments were accumulated in the Orbitrap until reaching $5e^4$ charges for a maximum of 120 ms.

SDS-PAGE for high-throughput slice-processing optimization

In order to facilitate higher-throughput for peptide preparation from gel-slices different 96-well membrane filter materials were compared to each other. This protocol optimization was performed by monitoring the yield for each different membrane filter materials (quantitative MS) and assessing whether the solvent conditions necessary for in gel-processing (100% ACN and MeOH) were compatible with the employed filter-material. The materials tested were 96 Well filter plates, AcroPrep 350ul, 1.0 μm Glass, natural (GF), AcroPrep, 350 μL, 0.2 μm wwPTFE membrane (wwPTFE 0.2), AcroPrep, 350 μL, 0.45 μm wwPTFE membrane (wwPTFE 0.45) and AcroPrep, 350 μL, 0.2 μm GHP membrane (GHP 0.2) against manual in-vial preparation in a receiver plate. As comparison, manual in-gel proteolysis protocol was performed, following the Protease MAX Surfactant (Promega) in gel digestion protocol.

For testing, equal amounts of 1000 ng, 500 ng, 250 ng, 100 ng of Bovine Serum Albumin (BSA) (Sigma Aldrich, A7906) were denatured with 3x Laemmli sample buffer in 10% 2-

Mercaptoethanol and incubated at 95 °C for 5 min. The samples were loaded on NuPAGE 4-12% Bis-Tris SDS-PAGE gels for gel-electrophoresis and then stained overnight in SimplyBlue™ SafeStain according to the manufactures protocol. For this experiment only the bands containing the monomeric and dimeric BSA were excised and transferred either to one of the filter-plates or to a receiver plate.

DDA data acquisition for high-throughput processing optimization

The BSA samples for optimizing the high-throughput filter-aided sample preparation were analyzed by LC-MS/MS on an LTQ-Orbitrap XL system (Thermo Fisher Scientific) equipped with an EASY nLC II system (Proxeon). Dried peptides were dissolved in 10 μL buffer A. For comparison of different filter plate materials 0.5 μL of the samples were injected (Supplementary Fig. 7A), whereas for the samples testing biases for hydrophobic/hydrophilic peptides and the dilution series samples 1 μL per single sample was injected (Supplementary Fig. 7B-D). Peptides were separated by reversed-phase chromatography over a 30 minutes gradient from 5-35% buffer B (95% ACN in 0.1% FA in H_2O) in Buffer A on the same column set-up previously described. The MS was operated in positive ion mode with up to 3 MS/MS spectra of the top 3 most-abundant precursor ions. The MS1 survey scan was performed in the Orbitrap between 350-1600 *m/z* and the fragment scan in the linear ion trap (OT/IT). The capillary voltage was set to 1.90 KeV and the capillary was heated to 200 °C. The top 3 most abundant precursors were fragmented by CID with a normalized collision energy of 35% and a minimal intensity of $2.5e^3$. Precursors having a charge between +2 and +4 were considered for fragmentation. MS1 resolution was fixed at 70'000 while the resolution for fragment ions was set at 15'000 at 400 *m/z*. Fragmented precursors were excluded from re-selection for 10 s. For MS2 scans an isolation width of 2 *m/z* with a maximum accumulation time of 250 ms were used.

Optimization of DIA method

To optimized cycle time and ion utilization in DIA-MS, we investigated the effect of different number of windows and resolution for the short 24 minutes LC-gradients. This optimization data was acquired on a Q Exactive^{TM} Plus Hybrid QuadrupoleOrbitrap^{TM} Mass Spectrometer (Thermo) interfaced with the EvosepOne system. As sample we tested 100, 500 and 1000 ng of in-solution digested HRK293 WT (ATCC collection) whole cell lysates, following a standard in-solution digestion (similar to [7]). Dilution series of digested full lysate was generated by diluting peptides in buffer A with 1:2500 (v/v) iRT peptides (Biognoysis) to a final loading volume of 100 μL. Following 10 minutes of sonication, peptides were loaded on Evotips. The C18 material of the Evotips was activated with 10 μL of Buffer B (98% acetonitrile and 0.1% formic acid in H_2O) and by soaking the tips in isopropanol. The tips were equilibrated by adding 10 μL of buffer A. Peptides were loaded by centrifugation at 300×*g* for 5 minutes. To prevent drying of the C18 material, 200 μL of Buffer A was added on top of each tip. Peptides were separated on a fused silica PicoTip^{TM} with an inner diameter of 100 μm (New Objective, Woburn, USA) and 50 μM tip diameter, in-house packed with 8 cm of C18 beads (MAGIC, 3 μm, 200 Å, Michrom BioResources, Auburn, USA). Peptides were separated using the '60 samples per day' method (24 minutes gradient) using the EvosepOne system. The MS was operated in positive mode with the capillary heated at 275 °C and kept at 2.5 KeV. For DIA we used 22 respectively 32 variable windows with +1 DA overlapping on the upper window boarder, ranging from 350 to 1650 *m/z*. The full MS1 scan was performed over a mass to charge range of 150 to 2000 *m/z* with a high-resolution of 70'000 fixed at 200 *m/z*. The AGC target was set to $3e^6$ with a maximum accumulation time set to 200 ms. For MS2 scans the resolution was fixed to 17'500 or 35'000 with an AGC target of $2e^5$ with HCD fragmentation in stepped mode employing a collisional energy of 25%, 27%, 30%, normalized to 500 *m/z* at charge state +1. Each MS2 scan time was set to 50 ms, leading to a maximum cycle time of 1.3 sec (Supplementary Fig. 8).

Data analysis of DIA-method optimization

MS raw files were analyzed with Spectronaut (version 13.12.200217.43655, Laika) against a human protein database (UniProtKB, downloaded on the 01.12.2019) and identification and quantitation was performed by directDIA. Analysis used default settings (BGS Factory settings) with minor adaptions (see DIA data analysis for DIP-MS data for exact parameters). For the dilution series of peptide loading of 100 ng, 500 ng, and 1000 ng, for the two lower dilution points only single injections with 22 and 32 windows at 17'500 and 32'000 MS2 resolution were acquired. For 1 ug samples triplicates with both window settings were acquired. For testing combinations of the two MS2 resolution settings versus the two different numbers of windows, at lower MS2 resolution triplicate injections, and for the higher resolution setting only 32 windows in duplicates were injected.

Data acquisition for absolute quantification of DIP-MS inputs

To perform quantification of the total amount of bait protein in DIP-MS input samples, an external calibration curve of heavy quant-peptide (SH-peptide, AADITSLYK containing isotope-labeled lysine) was generated. For the external calibration curve a total amount of 5000, 1000, 100 and 10 fmol of SH-peptide were spiked into tryptic digested BSA background (0.2 μg/μL). The DIP-MS input samples for PFDN2 (digested 1 μL of concentrated input samples of replicates one and two) and for the DIP-MS of UXT (digested 0.5 μL of concentrated input samples of replicate one) were diluted in 20 μL buffer A (2% ACN and 0.1% FA in H_2O). For all samples 2 μL were injected. The data acquisition method is described under data acquisition of reciprocal AP samples.

Data analysis for high-throughput processing optimization

MS1 based label-free quantification (LFQ) quantities were extracted with Skyline (version 20.1.0.76) [8]. The canonical BSA (Bovine Serum albumin, UniProtKB Identifier P02769) was *in silico* digested with PeptideCutter from ExPASy (http://web.expasy.org/peptide_cutter/) to

tryptic peptides (allowing for 1 misscleavage, with carboxymethyl-cysteine residues, C-term K/R, even before P) with a minimum peptide length of 5 aminoacids and peptides within a mass range of 250 to 3000 Dalton. From the theoretical 132 peptides, we extracted the precursor intensity for 57 peptides, while for the remaining peptides we observed sparse signals. In the Skyline transition settings tab the following parameters were used: MS1 counts, as Precursor mass analyzer Orbitrap with a resolving power of 60’000 at 400 *m/z*. The fragment mass to charge extraction range was set from 50 to 1500 *m/z* allowing for a 0.5 *m/z* ion match tolerance. Charge states for MS1 extraction was set between 2+ and 4+. To ensure confidence in selected peaks, the monoisotopic [M] and the [M+1] and [M+2] precursor signals were extracted. From Skyline MS1 LFQ values for all three charge states were exported. The data was further filtered in R (version 4.2.0) to the maximum peptide intensity across all samples and across charge states. The other 2 charge states were not considered within the analysis. The gravy index was obtained by a custom Perl script.

Data analysis for absolute quantification of bait proteins

The quantitative data were extracted with Skyline (version 20.1.0.76), by analysis of MS1 based LFQ values for the heavy and light SH-peptide (AADITSLYK) from the DIP-MS input samples. To achieve absolute quantitation of the SH-peptide, an external calibration curve of heavy quant-peptide [9] was generated and a linear regression model calculated. The calibration curve was used, to estimate the amount corresponding the independently measured SH-quant peptide intensities in our DIP-MS samples. Quantitative data were exported using the total MS1 area of the heavy SH-peptide. For LFQ values of the DIP-MS input samples the light version of the SH-peptide, which is the tryptic cleavage product of the tag of the bait proteins was extracted. Absolute bait concentration was calculated from the calibration curve employing the statistical software R (version 4.2.0). The derived linear regression of the heavy SH-quant peptide standards $y = 0.8258x + 8.6503$ ($R^2 = 0.96$) and the MW (PFDN2: 16’648 Da, and UXT: 18’246 Da) from UniProtKB [10] were used to derive

absolute amounts. Estimates of absolute bait input amounts were derived employing this formula:

$$bait\,[\mu g] = 10^{\frac{(Total\ Area\ MS1 - 8.6503)}{0.8258}} \times MW \ \times 10^{-9} \times Y_{Inj.Dil.Factor} \times Z_{Original\ Dil.Factor}$$

The *Original Dilution Factor* was the fraction separated for absolute quantification from the total DIP-MS inputs before loading on the native-PAGE. These final volumes varied, as the final concentration volume cannot be exactly standardized by filter assisted concentration. For PFDN2, replicate 1, an injection amount of 1 μL out of 40 μL, and for replicate 2, 1 μL out of 45 μL was used for absolute quantitation. For the UXT replicate 1 DIP-MS input, 0.5 μL out of 55 μL were used for quantitation of the absolute bait amount. The *Injection Dilution Factor* was for all three inputs 10 μL. The total Area MS1 can be found in Supplementary data 3.

Generation of fractionated GFP controls for interaction scoring

For scoring interaction partners, a set of six control GFP-affinity purifications were prepared as described in the method section and reported in previous study [11]. Following peptide clean-up, peptides were high-pH fractionated over a reversed-phase C18 spin-column (The Nest group) into seven fractions.

The fractions were acquired on an Orbitrap Elite mass spectrometer (Thermo Scientific) coupled to an Easy-nLC 1000 system (Thermo Scientific). Peptides were separated on a Thermo PepMap RSLC column (15 cm length, 75 μm inner diameter) with a 60 min gradient from 5% to 35% buffer B (95% ACN in 0.1% FA in H_2O) in buffer A (2% ACN and 0.1% FA in H_2O) at a flow rate of 300 nL/min. The mass spectrometer was operated in data-dependent acquisition (DDA) mode with the following parameters: one full FTMS scan (350-1600 *m/z*) at 120’000 resolution followed by 15 MS/MS scans in the Ion Trap. Charge states lower than two and higher than seven were rejected. Selected ions were isolated using a quadrupole mass filter of 2.0 *m/z* isolation window. Precursors with MS signal that exceeded a threshold of 500 were fragmented (CID, Normalized Collision Energy 35%). Selected ions were

dynamical excluded for 30 s Acquired spectra were searched using MaxQuant (version 1.5.2.8) embedded with the Andromeda search engine against human proteome reference dataset (UniProtKB, downloaded on 10.10.18) extended with reverse decoy sequences. The search parameters were set to include only full tryptic peptides, maximum one missed cleavage, carbamidomethyl as static peptide modification, oxidation (M) and phosphorylation (S, T, Y) as variable modification and "match between runs" option. The MS and MS/MS mass tolerance were set, respectively, to 4.5 ppm and 0.5 Da. False discovery rate of 1% was used at the protein level to infer the protein presence. The protein abundance was determined from the intensity of top two unique peptides for each protein. The 42 high-pH fractionated fractions were combined within MaxQuant applying the tab "fractionation". This resulted in a list of six GFP controls, with deep coverage of contaminants.

Manually curated list of reported PPIs and complexes of core-subunits

The first reference list is a literature curated list of 16 well characterized PFD and PFDL core complex subunits (Supplementary Table 1). A second larger reference list for comparison of the performance was generated covering all potential interactions and subsequent manual curation going back to the original publications. As source for the assignment of binary interactions, the interactions of all core-subunits of the PFD and PFDL as well as the PAQosome and CCT/TRiC were extracted from BioGRID [12] (July 2021, version 4.4.199) and IID [13] (July 2021, version 2018-11). This list covers 566 studies and covers 1892 reported PPIs (Supplementary Table 4). Complex-level information was extracted from CORUM [14], and literature [15] in order to derive complex identities (Supplementary Table 5).

Benchmarking of *PPI-prophet* against other co-fractionation tools

To benchmark the different co-fractionation MS software, we utilized Prince (https://github.com/fosterlab/PrInCE), EPIC (https://github.com/BaderLab/EPIC) and

PCprophet (https://github.com/anfoss/PCprophet). For *PPIprophet* analysis we utilized the output provided as Supplementary Data 2.

For Prince, it is necessary to utilize a gold standard dataset, and we utilized the gold standard dataset included in the package. The analysis was run utilizing the PrInCE wrapper function, with default parameters (min_points = 1, min_consecutive =5, min_pairs = 3, NA imputation and smoothing enabled, width of 4 and max_gaussian=5, with 10 fold cross validation).

We used the Docker version of EPIC with SVM classifier, using a custom PPI database derived from CORUM. All available features were calculated (PCCN/Jaccard/Apex/MI/Euclidean/WCC/Bayes/PCC).

For PCprophet, the CORUM database was used to FDR calculation, which was fixed at 10%. Default parameters were used (collapse_mode = GO) and the derive complexes were collapsed to PPIs using the assumption of full connectedness amongst all subunits.

To derive true positive interactions, we used the list of curated literature protein interactions (Supplementary Table 4), while for STRING we filtered using 0.6 combined score and the filter for protein IDs identified in the PFDN2 DIP-MS experiment to avoid mixing MS identification vs tool performance.

Orthogonal evidence of DIP-MS interactions by *in vivo* proximity interaction datasets

To validate the protein interactions partners found by DIP-Ms, a set of published in-vivo proximity ligation (BioID) datasets was used. The proximity interaction datasets were filtered to comprise only interactions for which the 16-core subunits of the PFD (PFDN1, PFDN2, VBP1, PFDN4, PFDN5, PFDN6), PFDL (ASDURF, PFDN1, PFDN6, PDRG1, UXT, URI1, POLR2E) and the PFDL containing PAQosome (PFDL subunits, WDR92, RPAP3, PIH1D1, RUVBL1, RUVBL2) were either used as baits or recovered as preys. As data sources the human cell map [16], a proximity interaction study on two PAQosome subunits [17], and all proximity interactions reported for core-subunits in BioGRID [12] were used. Further, for each

dataset the UniProtKB associations and gene names were manually curated to make the datasets comparable. If multiple UniProtKB associations were reported for one gene name, the UniProtKB which was reported in the other dataset was selected to maximize the overlap. The proximity interaction dataset of Cloutier et al. 2021 was filter to greater or equal log2FC of 2 and p-value of 0.01 resulting in 71 significant interactions for PIH1D1 and 78 significant interactions for the UXT BioID experiment respectively.

Protein complex structural predications

Structural prediction of alternative PDRG1 containing Prefoldin complex (PFD homolog, PFDh) were performed in ColabFold (version 1.3.0) [18], which combines MMseqs2 based multiple sequence alignment (MSAs) with AlphaFold2 [19] and AlphaFold-multimer [20] for protein complex structure prediction. Within ColabFold the MSAs were generated by MMseqs2 using the UniRef100 and environmental sequences. As MSA input the consensus sequences of all subunits obtained from UniProtKB were provided. As a template for the subunit order the deposited experimental structures of canonical PFD (PDB: 6NRD) [21] was used. The subunit order for PFDh complex and canonical PFD complex were the following: PFDN2:PFDN1:PFDN5:PFDN6:PFDN4/PDRG1:VBP1; and for PFDL: PFDN2:ASDURF:UXT:PFDN6:PDRG1:URI1(residue 24 to 222). For URI1 the disordered regions from residue 1 to 23 and residue 223 to 431 were removed from the URI1 prediction. For the MSA, the option for unpaired+paired setting was employed. Template mode was set to PDB 70. Advanced settings were sued with default parameters. The PFD and PFDh structures were relaxed (amber), but for PFDL the setting was not chosen due to time-outs during prediction. Five models were generated per complex, whereas they were ranked by the weighted score. Weighted scores were calculated by the addition of 0.8 x pTM with 0.2 x ipTM-score. Structures and structural alignments were visualized with ChimeraX (version 1.4) [22]. All modelled structures are deposited in GitHub at https://github.com/anfoss/PPIprophet.

Structural alignment of predicted structures

For binary structural alignments of ASDURF to other core-subunits, the tool US-align [23] (Version 20220511) was used (https://zhanggroup.org/US-align/). AlphaFold predicted structures were obtained from UniProtKB. The alignment was performed applying sequence independent structure alignment on the CA (protein) backbone of residues. TM-scores were normalized to the length of ASDURF, and the second protein, RMSD and alignment length. A threshold of > 0.5 on the average TM-score was used to estimate if the structure of ASDRUF shares same global topology with other core-subunits of the PFD, PFDL and PAQosome subunits. CCT/TRiC subunits alignment were used as negative controls. Alignments of predicted complex structures (PFD, PFDh and PFDL) were performed by multiple structure alignment (MSTA) using US-align with default parameters and a TM-cutoff of 0.45. As control an experimental canonical PFD structure (PDB: 6NRD) was used, although it lacked sufficient resolution.

Image source data for Supplementary Figures

A

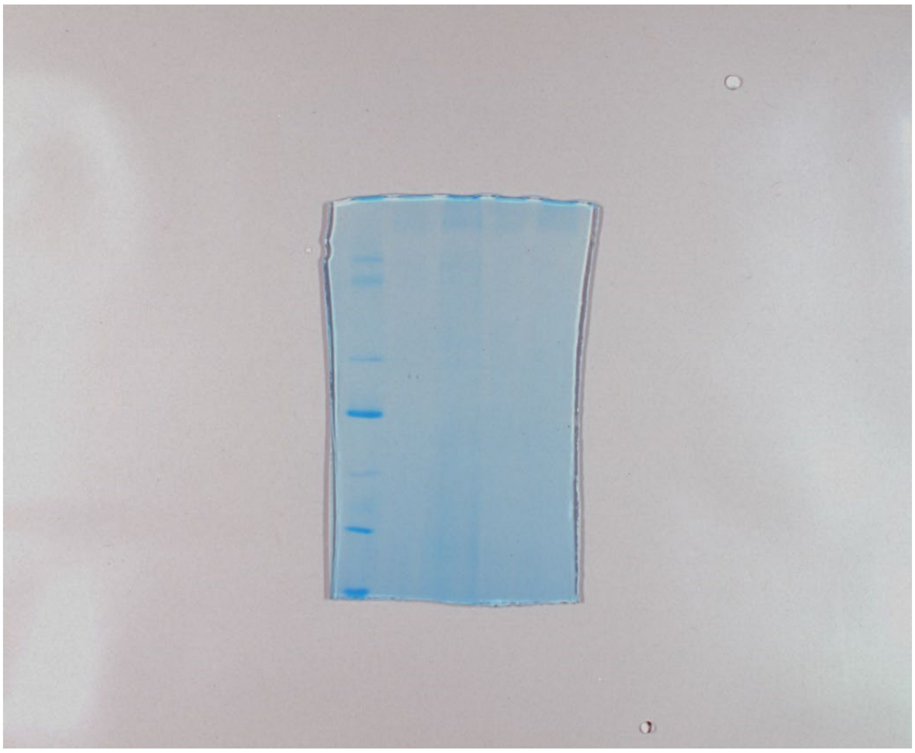

B

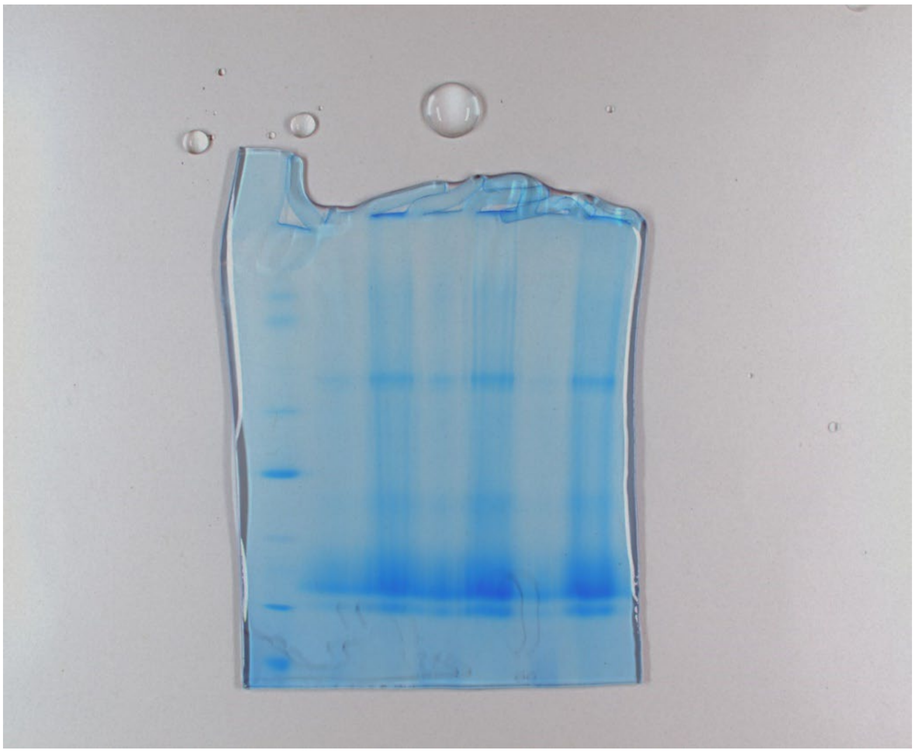

Image source data 1. Raw blue native-PAGE images of PFDN2 DIP-MS experiments. A. Uncropped and unprocessed BNP image of PFDN2 DIP-MS experiment. Lane 1 contains molecular weight standard. **B.** Uncropped and unprocessed BNP image of PFDN2 DIP-MS experiment. Lane 1 contains molecular weight standard.

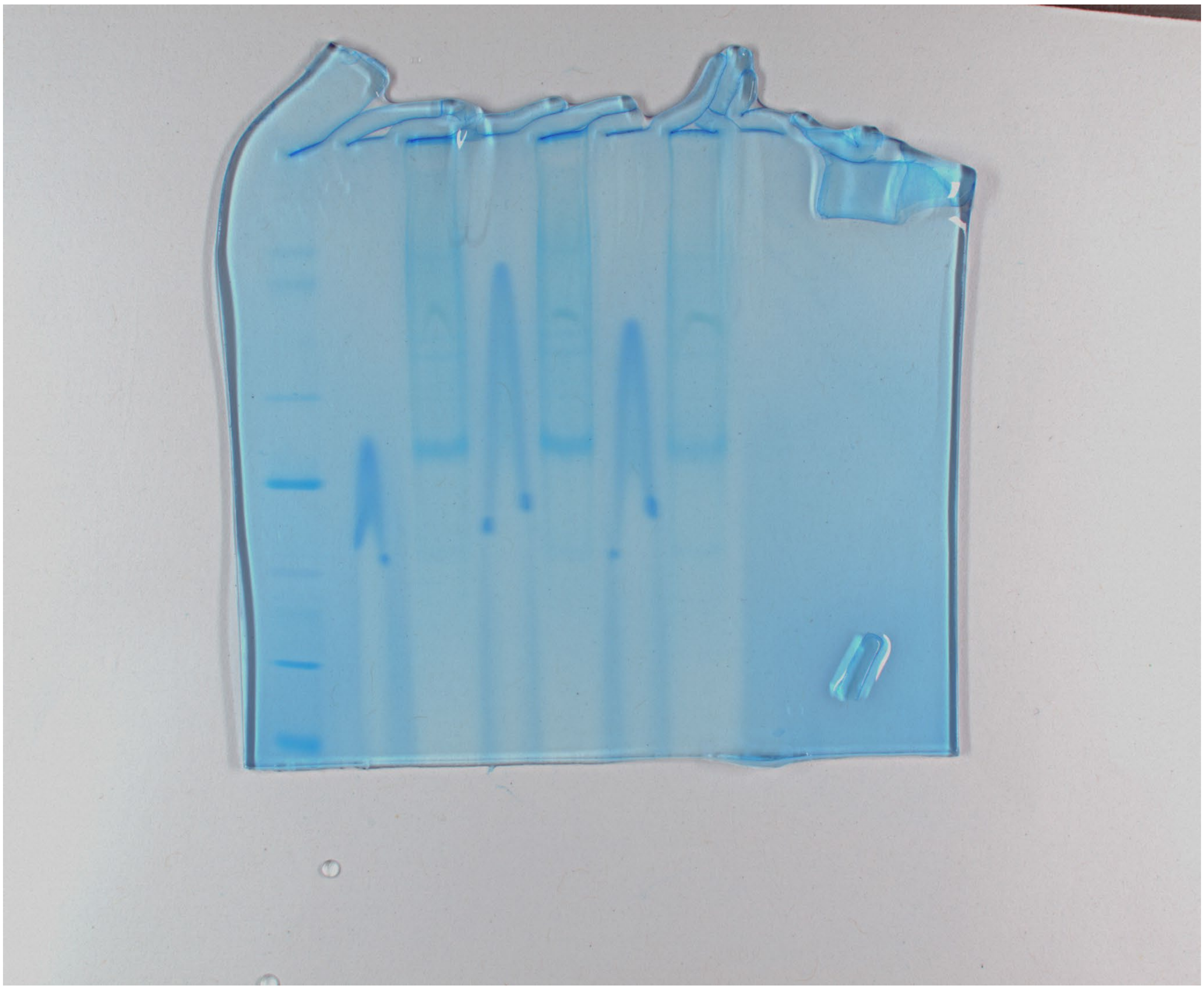

Image source data 2. Raw blue native-PAGE images of UXT DIP-MS experiments. Uncropped and unprocessed BNP image of UXT DIP-MS experiment. Lane 1 contains molecular weight standard.

References

1. Hu, G., et al., *flDPnn: Accurate intrinsic disorder prediction with putative propensities of disorder functions.* Nat Commun, 2021. **12**(1): p. 4438.
2. Gstaiger, M., et al., *Control of nutrient-sensitive transcription programs by the unconventional prefoldin URI.* Science, 2003. **302**(5648): p. 1208-1212.
3. Pinard, M., et al., *Unphosphorylated Form of the PAQosome Core Subunit RPAP3 Binds Ribosomal Preassembly Complexes to Modulate Ribosome Biogenesis.* J Proteome Res, 2022. **21**(4): p. 1073-1082.
4. Heusel, M., et al., *Complex-centric proteome profiling by SEC-SWATH-MS.* Molecular Systems Biology, 2019. **15**(1).
5. Skinnider, M.A. and L.J. Foster, *Meta-analysis defines principles for the design and analysis of co-fractionation mass spectrometry experiments.* Nature Methods, 2021. **18**(7): p. 806-+.
6. Glatter, T., et al., *An integrated workflow for charting the human interaction proteome: insights into the PP2A system.* Molecular Systems Biology, 2009. **5**.
7. Hauri, S., et al., *A High-Density Map for Navigating the Human Polycomb Complexome.* Cell Reports, 2016. **17**(2): p. 583-595.
8. Pino, L.K., et al., *The Skyline ecosystem: Informatics for quantitative mass spectrometry proteomics.* Mass Spectrometry Reviews, 2020. **39**(3): p. 229-244.
9. Wepf, A., et al., *Quantitative interaction proteomics using mass spectrometry.* Nature Methods, 2009. **6**(3): p. 203-205.
10. Bateman, A., et al., *UniProt: a worldwide hub of protein knowledge.* Nucleic Acids Research, 2019. **47**(D1): p. D506-D515.
11. Uliana, F., et al., *Phosphorylation-linked complex profiling identifies assemblies required for Hippo signal integration.* Molecular Systems Biology, 2023. **19**(4).
12. Stark, C., et al., *BioGRID: a general repository for interaction datasets.* Nucleic Acids Research, 2006. **34**: p. D535-D539.
13. Kotlyar, M., et al., *IID 2018 update: context-specific physical protein-protein interactions in human, model organisms and domesticated species.* Nucleic Acids Research, 2019. **47**(D1): p. D581-D589.
14. Giurgiu, M., et al., *CORUM: the comprehensive resource of mammalian protein complexes-2019.* Nucleic Acids Res, 2019. **47**(D1): p. D559-D563.
15. Lynham, J. and W.A. Houry, *The Multiple Functions of the PAQosome: An R2TP-and URI1 Prefoldin-Based Chaperone Complex.* Prefoldins: The New Chaperones, 2018. **1106**: p. 37-72.
16. Go, C.D., et al., *A proximity-dependent biotinylation map of a human cell.* Nature, 2021. **595**(7865): p. 120-+.
17. Cloutier, P., et al., *Upstream ORF-Encoded ASDURF Is a Novel Prefoldin-like Subunit of the PAQosome.* Journal of Proteome Research, 2020. **19**(1): p. 18-27.
18. Mirdita, M., et al., *ColabFold: making protein folding accessible to all.* Nat Methods, 2022. **19**(6): p. 679-682.
19. Jumper, J., et al., *Highly accurate protein structure prediction with AlphaFold.* Nature, 2021. **596**(7873): p. 583-589.
20. Evans, R., et al., *Protein complex prediction with AlphaFold-Multimer.* bioRxiv, 2022.
21. Gestaut, D., et al., *The Chaperonin TRiC/CCT Associates with Prefoldin through a Conserved Electrostatic Interface Essential for Cellular Proteostasis.* Cell, 2019. **177**(3): p. 751-+.
22. Pettersen, E.F., et al., *UCSF ChimeraX: Structure visualization for researchers, educators, and developers.* Protein Sci, 2021. **30**(1): p. 70-82.
23. Zhang, C., et al., *US-align: Universal Structure Alignments of Proteins, Nucleic Acids, and Macromolecular Complexes.* bioRxiv, 2022.
