# Supplementary material for: DIP-MS: ultra-deep interaction proteomics for the deconvolution of protein complexes
Source: Nat Methods. 2024 Mar 26;21(4):635–47. doi: 10.1038/s41592-024-02211-y (PMC11009110; doi:10.1038/s41592-024-02211-y)
Supplement: Supplementary file 2 — Reporting Summary [file 41592_2024_2211_MOESM2_ESM.pdf]

nature portfolio

Corresponding author(s): Fabian Frommelt and Matthias Gstaiger

Last updated by author(s): Jan 17, 2024

Reporting Summary

Nature Portfolio wishes to improve the reproducibility of the work that we publish. This form provides structure for consistency and transparency in reporting. For further information on Nature Portfolio policies, see our Editorial Policies and the Editorial Policy Checklist.

Statistics

For all statistical analyses, confirm that the following items are present in the figure legend, table legend, main text, or Methods section.

n/a	Confirmed	
☐	☒	The exact sample size (*n*) for each experimental group/condition, given as a discrete number and unit of measurement
☐	☒	A statement on whether measurements were taken from distinct samples or whether the same sample was measured repeatedly
☐	☒	The statistical test(s) used AND whether they are one- or two-sided<br>*Only common tests should be described solely by name; describe more complex techniques in the Methods section.*
☒	☐	A description of all covariates tested
☐	☒	A description of any assumptions or corrections, such as tests of normality and adjustment for multiple comparisons
☐	☒	A full description of the statistical parameters including central tendency (e.g. means) or other basic estimates (e.g. regression coefficient) AND variation (e.g. standard deviation) or associated estimates of uncertainty (e.g. confidence intervals)
☐	☒	For null hypothesis testing, the test statistic (e.g. *F*, *t*, *r*) with confidence intervals, effect sizes, degrees of freedom and *P* value noted<br>*Give P values as exact values whenever suitable.*
☒	☐	For Bayesian analysis, information on the choice of priors and Markov chain Monte Carlo settings
☒	☐	For hierarchical and complex designs, identification of the appropriate level for tests and full reporting of outcomes
☐	☒	Estimates of effect sizes (e.g. Cohen's *d*, Pearson's *r*), indicating how they were calculated

Our web collection on statistics for biologists contains articles on many of the points above.

Software and code

Policy information about availability of computer code

Data collection	Fusion FX6edge (VILBER Lourmat) version 18.02-SN<br>MaxQuant (Max-Planck-Institute for biochemistry) version 1.5.2.8<br>Skyline (MacCoss Lab Software) version 20.1.0.76<br>Spectronaut (Biognosys) version 13.12.200217.43655, Laika)
Data analysis	R (The R foundation) version R-3.6.1<br>Python (v3.7.2)<br>PPIprophet (https://github.com/anfoss/PPIprophet) v1<br>Clustal Omega (https://www.ebi.ac.uk/Tools/msa/clustalo/) v2.1<br>ColabFold (https://colab.research.google.com/github/sokrypton/ ColabFold/blob/v1.3.0/AlphaFold2.ipynb) version 1.3.0<br>Jalview https://www.jalview.org/ version 2.11.2.0<br>Cytoscape Version: 3.8.2<br>US-align (Zhang group; https://zhanggroup.org/US-align/) Version 20220511<br>flDPnn (http://biomine.cs.vcu.edu/servers/flDPnn/) Version: December2021<br>UCSF ChimeraX: Structure visualization for researchers, educators, and developers. (UCSF) version 1.4<br>PPIprophet was developed for this study and is freely accessible under https://github.com/anfoss/PPIprophet .

For manuscripts utilizing custom algorithms or software that are central to the research but not yet described in published literature, software must be made available to editors and reviewers. We strongly encourage code deposition in a community repository (e.g. GitHub). See the Nature Portfolio guidelines for submitting code & software for further information.

Data

Policy information about availability of data

All manuscripts must include a data availability statement. This statement should provide the following information, where applicable:

- Accession codes, unique identifiers, or web links for publicly available datasets
- A description of any restrictions on data availability
- For clinical datasets or third party data, please ensure that the statement adheres to our policy

The mass spectrometry proteomics data and Spectronaut, Skyline and MaxQuant outputs have been deposited to the ProteomeXchange Consortium via the PRIDE partner repository with the dataset identifier PXD035032. Human protein fasta files have been retrieved from UniProtKB (Taxonomic identifier 9606, status reviewed, downloaded on the 01.12.2019, https://www.uniprot.org/) and is deposited alongside the MS data. The with ColabFold (version 1.3.0.) predicted structural models, coelution data and PPIprophet parameters are deposited on Github https://github.com/anfoss/DIP-MS_data. PDB entries 2XSZ and 6NRD are accessible via https://www.rcsb.org/.

Human research participants

Policy information about studies involving human research participants and Sex and Gender in Research.

Reporting on sex and gender	n/a
Population characteristics	n/a
Recruitment	n/a
Ethics oversight	n/a

Note that full information on the approval of the study protocol must also be provided in the manuscript.

Field-specific reporting

Please select the one below that is the best fit for your research. If you are not sure, read the appropriate sections before making your selection.

☒ Life sciences ☐ Behavioural & social sciences ☐ Ecological, evolutionary & environmental sciences

For a reference copy of the document with all sections, see nature.com/documents/nr-reporting-summary-flat.pdf

Life sciences study design

All studies must disclose on these points even when the disclosure is negative.

Sample size	No prior sample size calculation was performed. Triplicate experiments were performed as routine in co-fractionation MS experiments.
Data exclusions	The WDR92-SH tagged AP-MS was excluded from the analysis due to low expression levels of the bait protein, resulting in low abundance and low sequence coverage.
Replication	All DIP-MS experiments (native co-fractionation dataset) were conducted in biological triplicates and the replications were successful. For all reciprocal AP-MS biological triplicates were performed, and all replicates were successful. As mentioned in the data exclusion section the AP-MS results of WDR92-SH were excluded due to low abundance and sequence coverage of the bait protein. For the estimation of the absolute amount of PFDN2 and UXT in the samples, external calibration curve and AP-inputs were injected once. Sample preparation optimization for different filter plates were either performed in triplicates or duplicates. DIA-method optimization was performed in triplicates.
Randomization	For sample preparation of PFDN2 and UXT DIP-MS fractions we optimized a 96-well format procedure with a randomization scheme. All samples have been LC-MS measured in sequential order for all experiments, including also the for the sample preparation randomized DIP-MS experiments. For AP-MS samples, no randomization was performed as standard in this type of experiments.
Blinding	Blinding was not performed due to the need to analyze samples sequentially and the randomization already performed at the sample preparation stage.

Reporting for specific materials, systems and methods

We require information from authors about some types of materials, experimental systems and methods used in many studies. Here, indicate whether each material, system or method listed is relevant to your study. If you are not sure if a list item applies to your research, read the appropriate section before selecting a response.

Materials & experimental systems

n/a	Involved in the study
☒	☐ Antibodies
☐	☒ Eukaryotic cell lines
☒	☐ Palaeontology and archaeology
☒	☐ Animals and other organisms
☒	☐ Clinical data
☒	☐ Dual use research of concern

Methods

n/a	Involved in the study
☒	☐ ChIP-seq
☒	☐ Flow cytometry
☒	☐ MRI-based neuroimaging

Eukaryotic cell lines

Policy information about cell lines and Sex and Gender in Research

Cell line source(s)	HEK293 WT cell line (Thermo Fisher Scientific), (Invitrogen) R70507<br>For cell line generation of the strep-HA expressing bait proteins the Flp-In HEK293 T-REx cells line (Thermo Fisher Scientific) (Invitrogen) R78007 were used.
Authentication	The cell lines were not authenticated.
Mycoplasma contamination	The cell lines were not tested for mycoplasma contamination.
Commonly misidentified lines (See ICLAC register)	No commonly misidentified cell lines were used in this study.
